# Intrahepatic Cholestasis of Pregnancy: An Autobiographical Case Report

**DOI:** 10.7759/cureus.21366

**Published:** 2022-01-18

**Authors:** Samantha Bartolone, Renee Alexis

**Affiliations:** 1 Osteopathic Medicine, Nova Southeastern University Dr. Kiran C. Patel College of Osteopathic Medicine, Davie, USA; 2 Obstetrics and Gynecology, Nova Southeastern University Dr. Kiran C. Patel College of Osteopathic Medicine, Davie, USA

**Keywords:** induction of labor, gestational disorder of the liver, intense pruritus, cholestatic liver disease, cholestasis of pregnancy

## Abstract

Intrahepatic cholestasis of pregnancy (ICP) is the most common liver disorder of pregnancy. This case report describes the author’s experience of being diagnosed with the condition, the course of treatment and outcome for her baby, and the emotional aspects of the disease.

## Introduction

Intrahepatic cholestasis of pregnancy (ICP) is the most common liver disorder associated with pregnancy; its occurrence varies by ethnicity and geographic region (0.1%-15.6%) [1]. ICP typically appears in the third trimester of pregnancy, although it can present much earlier in some women. The most common symptom present is pruritus, which varies in intensity in each individual patient. Other symptoms can include pain in the right upper quadrant, pale stool, and dark urine. Elevated serum bile acids are used to diagnose the disease. Other cholestatic laboratory values may be elevated, including aspartate aminotransferase, alanine transaminase, alkaline phosphatase, gamma-glutamyl transferase, and bilirubin. ICP increases the risk of stillbirth for the baby, as well as the likelihood of needing specialized care. In most women, pruritus resolves after delivery.

This case report describes the author’s experience of being diagnosed with ICP at 33 weeks of gestation and delivering the baby at 34 weeks and one day, along with the emotional implications of the experience. Throughout this case report, “I” will refer to myself, Samantha Bartolone. I believe that narrative medicine has great value and is a crucial part of holistic healthcare. It is my hope that this article will give readers a clear mental picture of what it feels like to experience ICP and prompt readers to seek more information about the condition.

## Case presentation

At almost 32 weeks pregnant, I was sitting on my living room couch, and a realization hit me - I had been scratching myself the entire day. I looked down, and my thighs and feet were red and excoriated. At age 28, this was my first pregnancy, and so far, it was uncomplicated and “easy.” I had not started using any new detergents, and I had no history of allergies. I quickly researched the different causes of pruritus in pregnancy. Fully expecting to be reassured that this was something normal and benign due to hormonal changes, I was instead dismayed by what I found. I found that “intrahepatic cholestasis of pregnancy” presents with pruritus and is associated with an increased risk of stillbirth. I panicked and contemplated going to the hospital that night but decided to call my doctor’s office first thing in the morning instead.

The next morning, my doctor was able to see me for an early appointment due to my concerns. I was first seen by a family medicine intern, who very empathetically listened and took my complaint seriously. He relayed my concerns to my doctor who requested that serum bile acid levels be drawn.

For the next week awaiting those laboratory results, I continued to itch. If I could rate the itch on a scale from one to 10, I would say it fluctuated between five and seven. I had itching all over my body, including deep in my ears and on my scalp. It took me longer than normal to fall asleep, and at times, I woke up realizing that I was scratching myself in my sleep. I kept myself busy with my studies, which helped keep my mind occupied, but I also spent a significant amount of time reading about ICP online.

Throughout the week of waiting, I began to be hyperaware of my baby’s fetal movements. The tragic stories that I read online started to occupy my mind to a degree that made me very anxious. Midway through the week, I became so anxious that I went to the hospital for an ultrasound. The ultrasound showed no abnormalities and a normal heart rate. I was so relieved.

Exactly one week later, I finally received a call from my doctor, informing me that I had ICP. My total serum bile acid level was 328.5 μmol/L (reference: <10 μmol/L). Ursodeoxycholic acid (UDCA) 300 milligrams was prescribed, three times daily with meals. I was advised to go to the hospital for further monitoring.

Over the next couple of days, I wore the fetal heart rate monitor and went through a series of tests, including urinalysis, complete blood count, comprehensive metabolic panel, and liver ultrasound. My baby was monitored with an ultrasound biophysical profile. All laboratory values and imaging were within normal limits, except for aspartate aminotransferase, alanine transaminase, alkaline phosphatase, and direct bilirubin, which were all elevated. I continued to take UDCA three times daily with meals and experienced no side effects. The itching remained at a five to seven in intensity. I was only 33 weeks and two days pregnant at this time, and my doctors wanted to wait until 34 weeks, an important milestone in fetal development, for induction. A steroid shot was administered to help my baby’s lungs develop in anticipation of the induction. At one point, my doctor came to speak with me and asked if I had any questions. I asked him, “If my baby were to be affected by this, and the worst were to happen, would there be a warning sign on the fetal heart monitor, you know, to catch it before it happens and intervene?” He replied, “No, the baby’s heart would stop, and there would be nothing we could do at that point.”

Since my baby and I were stable, I was discharged from the hospital and advised to await a call from my doctor for further directions on my induction later in the week. The days of waiting felt like a blur; my mother came to keep my mind occupied and prevent the words of my doctor from creeping into my thoughts.

**Figure 1:**
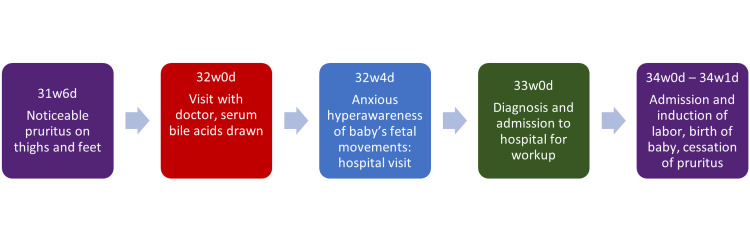
Timeline summary of the case “#w” and “#d” refer to weeks and days of gestation. The image was made by the author using the 2017 version of PowerPoint.

Four days later, I was advised by my doctor to come to the hospital to deliver my baby via induction. I had uneventful labor, and my son was born about 36 hours later. He weighed five pounds and two ounces. The itching that I had been experiencing for the past two weeks disappeared completely after delivery. After speaking with my family about my experience, I learned that my paternal aunt had ICP with both of her pregnancies. A simplified timeline is illustrated in Figure 1.

After my son was delivered, he was taken to the neonatal intensive care unit (NICU) and placed on continuous positive airway pressure (CPAP) initially; however, he was able to breathe effortlessly on his own within the week. He also received phototherapy for a few days due to neonatal jaundice. In total, his NICU stay was extended to one month due to bradycardia of prematurity but was otherwise uneventful.

## Discussion

The itch

For most women with ICP, pruritus is the first and most obvious symptom of the disease. Interestingly, the severity of pruritus does not correlate with the serum bile acid level as much as it does with serum lysophosphatidic acid and autotaxin [1]. For example, my bile acid level would be considered extremely elevated, but my itch level was moderate. According to research, UDCA is the most effective at relieving pruritus; however, some women experience pruritus that is refractory to UDCA and need to try other methods such as S-adenyl-methionine, cholestyramine, rifampin, hydroxyzine, or menthol lotions, to name a few [2]. For me, a cold shower and aloe with menthol provided enough relief.

Bile acid testing

According to current guidelines, ICP can be diagnosed when serum bile acid levels are above 10 μmol/L. The risk for adverse outcomes is increased when serum bile acids reach 40 μmol/L [3,4]. These risks are further increased, with a 3.44% risk of stillbirth, when that number reaches 100 μmol/L [5].

The fact that it took a week for my serum bile acid levels to come back did not affect the outcome of my pregnancy. However, you can imagine that if you started to itch later in pregnancy, for example, at 37 weeks, waiting a week to be diagnosed could put your baby at risk. Likewise, it is possible for itching to precede an elevated serum bile acid level, necessitating repeated testing to achieve a diagnosis [6]. For this reason, a faster turnaround time for bile acid testing in laboratories is important to achieve. Judd’s Legacy, a nonprofit organization, is currently making great strides toward this goal in South Carolina [7].

Treatment

Since the risk of stillbirth due to ICP increases in the last weeks of pregnancy [8,9], the current treatment is early delivery and UDCA two to three times daily [2]. Because my bile acid levels were well above 100 μmol/L, I was eager to be induced. Knowledge of this increased risk was very frightening; because of this fear, I wanted to deliver my son sooner than 34 weeks. My doctors did the right thing for both my son and me, however, by extending my induction date; this graduated my baby from “moderately preterm” status (32-34 weeks) to “late preterm” status (34-36 weeks) [10].

Psychological implications and post-ICP concerns

Being diagnosed with a “rare condition” late in pregnancy comes as a shock to those of us who experience it. Along with the symptoms and diagnosis comes more questions. For example, why did this happen? Researchers have identified some risk factors for ICP, such as two adenosine triphosphate-binding cassette genes, ABCB4 and ABCB11 [11]. There is no doubt in my mind that I was genetically predisposed to this, as evidenced by my aunt experiencing it with both of her pregnancies. Other risk factors include age, ethnicity, and multiple gestation pregnancies [12]. In addition, some women are made aware of underlying liver pathologies only after experiencing ICP [13], causing women to wonder if there are additional health problems to be faced in the future.

The psychological impact of ICP has not been investigated to my knowledge, but I have witnessed it firsthand and through participating in online support groups. Some sources of psychological stress include the itch itself, the lack of sleep associated with the itch, the anxiety of waiting to be diagnosed, and the anticipation of delivering the baby safely. The stress does not end after the baby is delivered, since there is the question of whether a premature birth or cholestasis itself will affect the baby’s long-term health. Most importantly, many moms are facing unimaginable grief after losing one or more babies to ICP.

Implications for the baby

It is well known that delivering a baby prematurely for any reason increases certain risks, such as respiratory distress syndrome and feeding difficulties. Some research demonstrates that the risks associated with prematurity are increased for babies born from mothers with ICP, even if born at term [14]. Concerning the possibility of other long-term effects for children born to mothers with ICP, one study did find a connection between maternal cholestasis and metabolic disease in offspring [15]. There is currently no evidence of other long-term complications.

## Conclusions

Experiencing intrahepatic cholestasis of pregnancy has taught me to expect the unexpected. I have a great appreciation for my doctors who listened to my concerns and took them seriously. Through online support groups, I have learned that these concerns are often dismissed. This leaves many women feeling anxious and unsupported during a very vulnerable time. It is very important to raise awareness of intrahepatic cholestasis of pregnancy among friends, family, and colleagues in medicine. Itching in pregnancy should not be considered “normal,” serum bile acid results should be obtained quickly, and proper evidence-based treatment should be given to all women with ICP. Sensitivity of providers toward the unique stresses that women with ICP face is also very important. I hope that this autobiographical case report has been informative and piqued further interest in learning about ICP.
